# Supplementary material for: Textual interpretation of transient image classifications from large language models
Source: Nat Astron. 2025 Oct 8;9(12):1869–78. doi: 10.1038/s41550-025-02670-z (PMC12708355; doi:10.1038/s41550-025-02670-z)
Supplement: Supplementary file 1 — Supplementary Figs. 1–3. [file 41550_2025_2670_MOESM1_ESM.pdf]

nature astronomy

Supplementary information https://doi.org/10.1038/s41550-025-02670-z

Textual interpretation of transient image classifications from large language models

In the format provided by the authors and unedited

Supplementary information

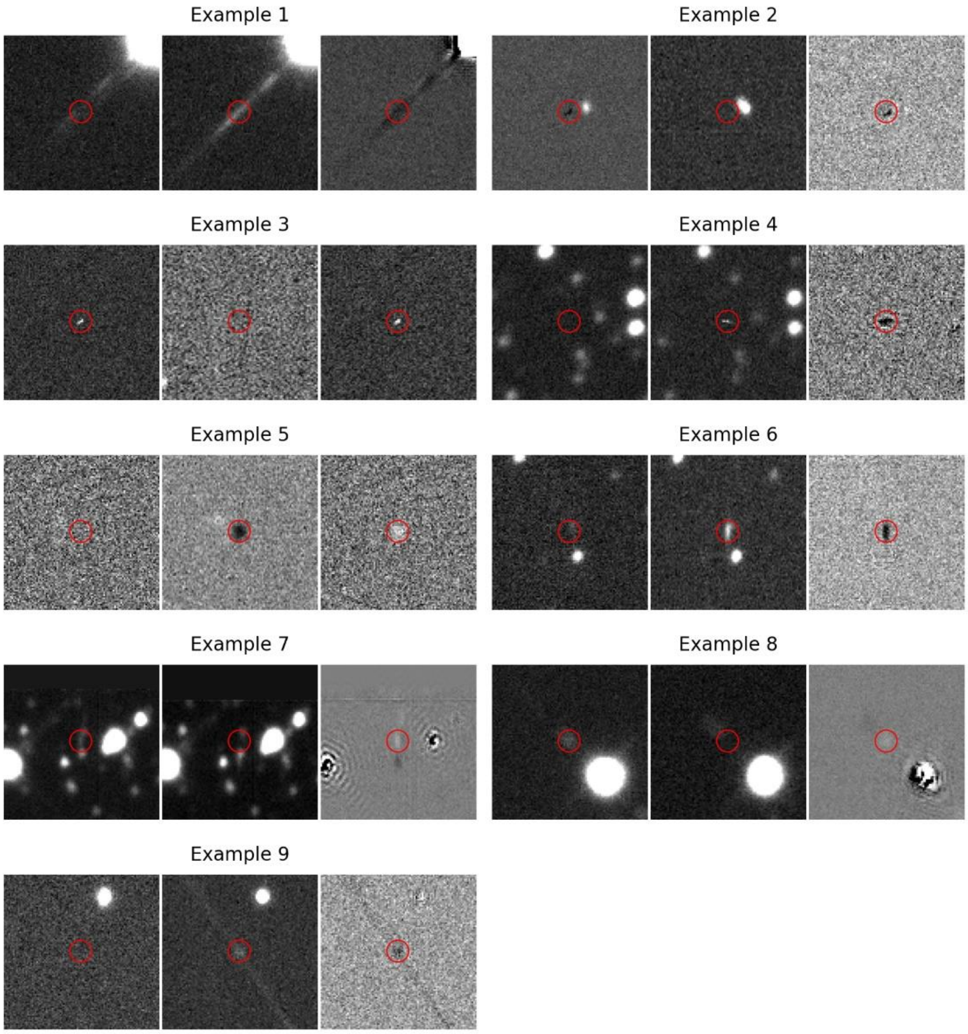

Supplementary Figure 1: Representative bogus examples used in Gemini's few-shot learning setup. Image triplets (New, Reference, Difference) are shown for 9 transients from the MeerLICHT dataset, all labeled as **Bogus**. These examples were included among the 15 annotated prompts used to guide Gemini's classification behavior. Descriptions for each are provided below.

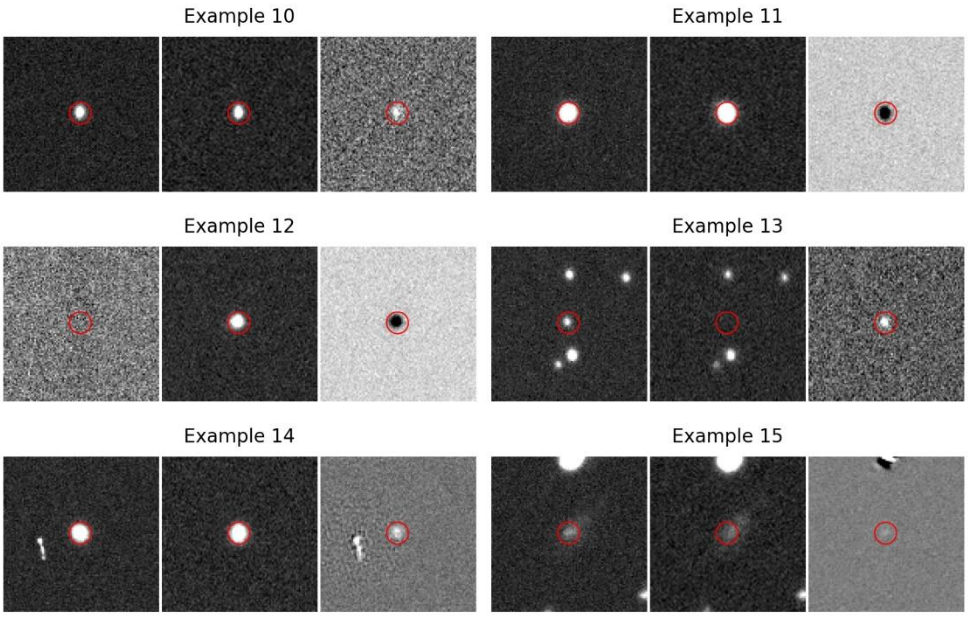

Supplementary Figure 2: Representative real examples used in Gemini's few-shot learning setup. Image triplets (New, Reference, Difference) are shown for 6 transients from the MeerLICHT dataset, all labeled as **Real**. These examples were included among the 15 annotated prompts used to guide Gemini's classification behavior. Descriptions for each are provided below.

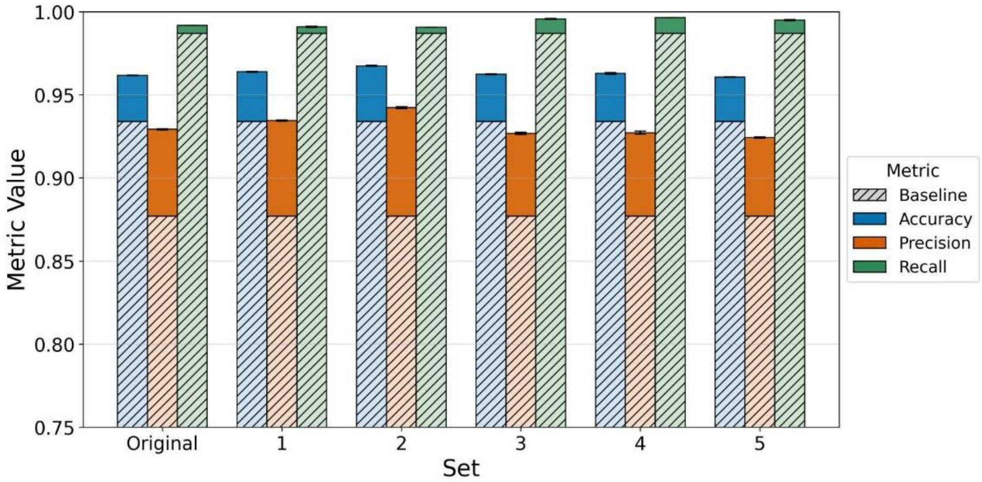

Supplementary Figure 3: Classification metrics for the original and five new guide-set prompts. Each group of bars shows the mean accuracy (blue), precision (red), and recall (green) over five Gemini runs; error bars represent the corresponding standard deviations, which are too small to be visible in most cases. The hatched lower segments in the bars mark the baseline metrics obtained in the December 2024 analysis, allowing direct visual comparison with the updated results.
